# Subclinical atherosclerosis and impaired bone health in patients with primary Sjogren’s syndrome: prevalence, clinical and laboratory associations

**DOI:** 10.1186/s13075-015-0613-6

**Published:** 2015-04-11

**Authors:** Fotini Gravani, Ioanna Papadaki, Eleni Antypa, Andrianos Nezos, Kyriaki Masselou, Dimitrios Ioakeimidis, Michael Koutsilieris, Haralampos M Moutsopoulos, Clio P Mavragani

**Affiliations:** Department of Rheumatology, General Hospital of Athens “G.Gennimatas”, Athens, Greece; Department of Pathophysiology, School of Medicine, University of Athens, Athens, Greece; Department of Radiology, General Hospital of Athens “G.Gennimatas”, Athens, Greece; Department of Physiology, School of Medicine, University of Athens, M. Asias 75, Athens, 11527 Greece; Department of Immunology, General Hospital of Athens “G. Gennimatas”, Athens, Greece

## Abstract

**Introduction:**

To determine the prevalence and clinical/laboratory associations of subclinical atherosclerosis and impaired bone health in primary Sjogren’s syndrome (SS).

**Methods:**

64 consecutive patients with primary SS, 77 with rheumatoid arthritis (RA) and 60 healthy controls (HC) οf similar age and sex distribution were enrolled. Demographics, clinical/laboratory features, classical risk factors for atherosclerosis and osteoporosis (OP) were recorded. Intima-medial thickness scores (IMT) and carotid/femoral (C/F) plaque formation, as well as bone mineral density (BMD) and fractures were evaluated. Determinants of IMT/BMD levels and the presence of plaque were assessed by univariate and multivariate models. Serum levels of the Wnt signaling mediators Dickkopf-related protein 1(DKK1) and sclerostin were determined in primary SS patients and HC.

**Results:**

Increased arterial wall thickening (IMT > 0.90 mm) and impaired bone health (defined as OP or osteopenia), were detected in approximately two-thirds of primary SS and RA patients, with a mean IMT value being significantly increased compared to HC. The presence of primary SS emerged as an independent risk factor for arterial wall thickening when traditional risk factors for cardiovascular disease (CVD) including age, sex, hypertension, smoking (pack/years), LDL and HDL levels were taken into account in a multivariate model [adjusted OR 95% (CI): 2.8 (1.04-7.54)]. In primary SS, age was revealed as independent predictor of increased IMT scores; age and lymphopenia as well as increased urine pH as independent determinants of C/F plaque formation and OP/osteopenia, respectively. An independent association of OP/osteopenia with plaque formation was observed when independent predictors for both variables were considered, with low DKK1 levels being associated with both plaque formation and lower BMD levels.

**Conclusions:**

Comorbidities such as subclinical atherosclerosis and impaired bone health occur frequently in primary SS, in association with disease related features and traditional risk factors. Wnt signaling mediators are potentially involved in the pathogenesis of both entities.

**Electronic supplementary material:**

The online version of this article (doi:10.1186/s13075-015-0613-6) contains supplementary material, which is available to authorized users.

## Introduction

Increased rates of subclinical atherosclerosis have been previously described in systemic autoimmune diseases, particularly in systemic lupus erythematosous (SLE) and rheumatoid arthritis (RA) [1-4]. However, traditional cardiovascular risk factors such as smoking, dyslipidemia, diabetes mellitus (DM), hypertension (HT) and increased body mass index (BMI), do not fully account for the high rates of subclinical atherosclerosis in these patients [5-7]. Additionally, osteopenia and osteoporosis (OP) rates, which have been found to be associated with surrogate markers of cardiovascular comorbidity in healthy populations [8], have been also shown to be increased in patients with SLE, RA and systemic sclerosis [9-11].

**Table 1:** **Prevalence of comorbidities in patients with primary SS or RA and in age- and sex-matched healthy individuals**

	**SS (n = 64)**	**RA (n = 77)**	**HC (n = 60)**	***P*** -value*	***P*** -value**	***P*** -value***
**Arterial wall thickening (IMT >0.90 mm), %**	59.4	63.8^a^	40.4	ns	<0.05	<0.01
**IMT levels, mean ± SD**	1.0 ± 0.3	1.0 ± 0.4^a^	0.9 ± 0.2	ns	<0.05	<0.01
**Presence of plaque, %**	68.8	84.4	56.9	<0.05	ns	<0.001
**Osteopenia, %**	51.6	54.2^a^	36.8	ns	ns	ns
**Osteoporosis, %**	7.8	18.1^a^	14.0	ns	ns	ns
**Osteoporosis/osteopenia, %**	59.4	72.2^a^	50.9	ns	ns	<0.05
**Fractures, %**	12.5	23.9^a^	8.5	ns	ns	<0.05

*Primary Sjogren's syndrome (SS) versus rheumatoid arthritis (RA); ** primary SS versus healthy controls (HC); *** RA versus HC; ^a^analysis based on 72 patients with RA. IMT, intima media thickness; ns, not significant.

**Table 2:** **Traditional and disease-related predictors of arterial wall thickening in patients with primary SS**

	**Univariate analysis**			**Multivariate analysis**	
	**IMT >0.90 (n = 38)**	**IMT ≤0.90 (n = 26)**	***P*** **-value**	**Odds ratio (95% CI)**	***P*** **-value**
**Traditional risk factors**					
Age, years	62.5 ± 9.2	49.8 ± 12.6	<0.001	1.1 (1.1, 1.2)	0.002
Female, %	92.1	96.2	ns		
Past medical history of CVD, %	7.9	3.8	ns		
Family history of CVD, %	13.2	15.4	ns		
Smoking, packs/year	10.5 ± 20.7	3.6 ± 7.4	ns		
Body mass index	29.0 ± 5.7	25.0 ± 4.4	0.02		
Diabetes, %	5.3	7.7	ns		
Hypertension, %	47.4	19.2	0.04		
Cholesterol levels, mg/dl	202.2 ± 35.2	183.8 ± 30.0	ns		
High-density lipoprotein, mg/dl	57.0 ± 13.9	53.5 ± 15.5	ns		
Low-density lipoprotein, mg/dl	126.8 ± 32.2	111.2 ± 25.0	0.03		
Triglycerides, mg/dl	111.5 ± 44.7	92.5 ± 38.8	ns		
Homocysteine levels, μmol/l	14.6 ± 4.6	14.2 ± 6.0	ns		
Uric acid, mg/dl	4.4 ± 1.1	3.6 ± 1.2	0.006		
Current steroid dose, mg	1.3 ± 2.3	1.1 ± 2.1	ns		
Total steroid dose, g	7.8 ± 17	6.6 ± 11.3	ns		
Current TSH levels, μ ΙU/dl	1.5 ± 1.4	1.8 ± 1.3	ns		
C-reactive protein, mg/l	3.1 ± 3.2	6.7 ± 16.0	ns		
Fibrinogen, mg/dl	416.6 ± 108.2	395.6 ± 128.1	ns		
25-hydroxy vitamin D3, ng/ml	22.3 ± 13.4	19.8 ± 7.6	ns		
**Disease-related features**					
Disease duration, years	8.7 ± 7.4	8.0 ± 6.6	ns		
SS disease activity index	1.8 ± 1.5	1.8 ± 1.7	ns		
Focus score, number of foci/4 mm^2^	2.6 ± 2.2	1.6 ± 1.4	ns		
Whole salivary flow, ml/15 minutes	2.0 ± 2.8	2.6 ± 3.1	ns		
Peri-epithelial disease, %	31.6	3.8	0.007		
Arthritis, %	23.6	46.1	ns		
Arthralgias, %	65.7	76.9	ns		
Dry mouth, %	84.2	76.9	ns		
Dry eyes, %	86.8	88.5	ns		
Salivary gland enlargement, %	21.1	15.3	ns		
Raynaud’s phenomenon, %	28.9	23.1	ns		
Lymphadenopathy, %	18.4	19.2	ns		
Splenomegaly, %	2.6	3.8	ns		
Palpable purpura, %	10.5	3.8	ns		
Monoclonal gammopathy (%)	10.5	3.8	ns		
Cryoglobulins (%)	2.6	0	ns		
Lymphoma (%)	18.4	7.6	ns		
Antinuclear antibodies >1/160, %	89.4	96.1	ns		
Anti-Ro/SSA, %	76.3	73.1	ns		
Anti-La/SSB, %	42.1	57.7	ns		
Rheumatoid factor titers, IU/ml	61.0 ± 104	58.3 ± 84.0	ns		
Complement 3, mg/ml	1.2 ± 0.2	1.1 ± 0.2	0.002		
Complement 4, mg/ml	0.2 ± 0.1	0.2 ± 0.1	ns		
Absolute number of WBC/mm^3^	5,647 ± 1903	5,727 ± 1785	ns		
Absolute number of lymphocytes/mm^3^	1,518 ± 463	1,645 ± 756	ns		
Erythrocyte sedimentation rate	34 ± 21	31 ± 22	ns		
γ-globulins, %	18.5 ± 5.2	18.6 ± 3.4	ns		
Urine specific gravity	1,013 ± 6	1,018 ± 8.0	0.007		
Urine pH	6.1 ± 0.7	6.1 ± 0.6	ns		

IMT, intima media thickness; SS, Sjogren’s syndrome; IMT, Intima media thickness; CVD, cardiovascular disease; TSH, thyroid stimulating hormone; WBC, white blood cells; ns, not significant.

**Table 3:** **Traditional and disease-related predictors of plaque formation in patients with primary SS**

	**Univariate analysis**			**Multivariate analysis**	
	**Plaque (n = 44)**	**No plaque (n = 20)**	***P*** **-value**	**Odds ratio (95% CI)**	***P*** **-value**
**Traditional risk factors**					
Age, years	60.1 ± 9.1	51.0 ± 16.2	0.006	1.1 (1.0, 1.1)	0.09
Female, %	93.2	95	ns		
Past medical history of CVD, %	6.8	5	ns		
Family history of CVD, %	18.2	5	ns		
Smoking, packs/year	9.1 ± 19.3	4.7 ± 9.3	ns		
Body mass index	27.6 ± 5.6	26.8 ± 5.5	ns		
Diabetes, %	9.1	0	ns		
Hypertension, %	38.6	30.0	ns		
Cholesterol levels, mg/dl	194.4 ± 33.9	194.9 ± 35.7	ns		
High-density lipoprotein, mg/dl	56.6 ± 15.1	51.2 ± 12.8	ns		
Low-density lipoprotein, mg/dl	116.1 ± 30.6	130.2 ± 27.7	ns		
Triglycerides, mg/dl	107.8 ± 43.9	93.9 ± 40.8	ns		
Homocysteine levels, μmol/l	15.3 ± 5.5	12.4 ± 3.8	ns		
Uric acid, mg/dl	4.1 ± 1.3	4.0 ± 0.9	ns		
Current steroid dose, mg	1.3 ± 2.2	1.1 ± 2.2	ns		
Total steroid dose, g	9.3 ± 17.5	3.7 ± 6.3	ns		
Current TSH levels, μ ΙU/dl	1.6 ± 1.5	1.6 ± 1.2	ns		
C-reactive protein, mg/l	3.2 ± 5.8	7.6 ± 17.0	ns		
Fibrinogen, mg/dl	410.0 ± 99.7	402.8 ± 151.2	ns		
25-hydroxy vitamin D3, ng/ml	21.1 ± 12.9	21.7 ± 7.0	ns		
**Disease-related features**					
Disease duration, years	8.9 ± 7.3	7.3 ± 6.6	ns		
SS disease activity index	1.7 ± 1.5	2.1 ± 1.8	ns		
Focus score, number of foci/4 mm^2^	2.7 ± 1.9	1.2 ± 1.4	0.04		
Whole salivary flow, ml/15 minutes	1.9 ± 2.8	3.0 ± 3.2	0.04		
Peri-epithelial disease, %	22.7	15.0	ns		
Arthritis, %	29.5	40.0	ns		
Arthralgias, %	68.2	75.0	ns		
Dry mouth, %	84.1	75.0	ns		
Dry eyes, %	90.9	80.0	ns		
Salivary gland enlargement, %	11.4	20	ns		
Raynaud’s phenomenon, %	34.1	10.0	0.04		
Lymphadenopathy, %	18.2	20.0	ns		
Splenomegaly, %	2.3	5	ns		
Palpable purpura, %	9.1	5	ns		
Cryoglobulins (%)	2.3	0	ns		
Lymphoma (%)	13.6	15.0	ns		
Antinuclear antibodies >1/160, %	86.4	95	ns		
Anti-Ro/SSA, %	72.7	75	ns		
Anti-La/SSB, %	47.7	50.0	ns		
Rheumatoid factor titers, IU/ml	53.7 ± 88.7	72.5 ± 108.6	ns		
Complement 3, mg/ml	1.1 ± 0.2	1.2 ± 0.3	ns		
Complement 4, mg/ml	0.2 ± 0.1	0.2 ± 0.1	ns		
Absolute number of WBC/mm^3^	5,533 ± 1968	6,006 ± 1516	ns		
Absolute number of lymphocytes/mm^3^	1,411 ± 476	1,924 ± 705	0.001	0.9 (0.9, 1.0)	0.02
Erythrocyte sedimentation rate	33 ± 21	31 ± 21	ns		
γ-globulins, %	18.3 ± 5.1	19.0 ± 2.7	ns		
Urine specific gravity	1,014 ± 6	1,016 ± 9	ns		
Urine pH	6.1 ± 0.7	6.1 ± 0.5	ns		

IMT, intima media thickness; SS, Sjogren’s syndrome; IMT, Intima media thickness; CVD, cardiovascular disease; TSH, thyroid stimulating hormone; WBC, white blood cells; ns, not significant.

**Table 4:** **Determinants of osteoporosis (OP) or osteopenia in patients with primary SS**

	**Univariate analysis**			**Multivariate analysis**	
	**Presence of osteoporosis or osteopenia (n = 38)**	**Absence of osteoporosis or osteopenia (n = 24)**	***P*** **-value**	**Odds ratio 95% (CI)**	***P*** **-value**
**Classical risk factors for osteoporosis**					
Age, years	59. 9 ± 10.1	52.8 ± 14.6	0.03		
Family history of fracture, %	5.4	27.2	0.02		
Past medical history of fracture, %	15	8.3	ns		
Body mass index	26.9 ± 4.7	28.2 ± 6.8	ns		
Age of menarche, years	13.4 ± 1.7	13.1 ± 2.4	ns		
Age of menopause, years	49.4 ± 4.1	50.6 ± 6.3	ns		
Calcium, mg/dl	9.7 ± 0.5	9.3 ± 0.6	0.02	3.2 (1.01, 10.4)	<0.05
Phosphate, mg/dl	3.4 ± 0.5	3.2 ± 0.6	ns		
Calcium urine/24 h, g	0.1 ± 0.1	0.1 ± 0.2	ns		
Phosphate urine/24 h, g	0.5 ± 0.2	0.6 ± 0.2	ns		
25-hydroxy-vitamin D3, ng/ml	21.2 ± 12.9	21.4 ± 8.1	ns		
Parathyroid hormone, pg/ml	50.2 ± 22.6	41.6 ± 16.6	ns		
Current thyroid stimulating hormone levels (μ IU/dl)	1.34 ± 2.1	1.3 ± 1.5	ns		
Total steroid dose, g	9.8 ± 17.8	3.2 ± 0.5	ns		
Current steroid dose, mg/day	1.6 ± 2.4	0.5 ± 1.4	ns		
**Disease-related features**					
Sjogren's syndrome activity index	1.85 ± 1.49	1.74 ± 1.8	ns		
Disease duration, years	9.1 ± 7.4	7.3 ± 6.3	ns		
Focus score, number of lymphocytic foci/4 mm^2^	1.9 ± 1.8	2.4 ± 1.9	ns		
Whole salivary flow, ml/15 minutes	2.0 ± 2.9	2.6 ± 3.1	ns		
Urine pH	6.3 ± 7.4	5.8 ± 0.4	0.03	4.5 (1.004, 20.4)	<0.05
Urine specific gravity	1,014 ± 6.9	1,015 ± 7	ns		
Arthralgias, %	80	54.2	0.03		
Peri-epithelial disease, %	27.5	8.3	ns		

ns, not significant.

**Figure 1:**
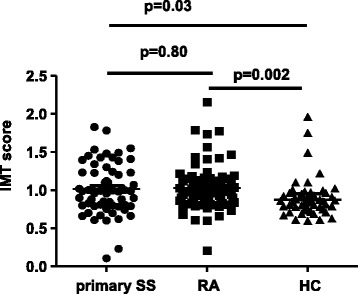
Intima media thickness (IMT) scores in patients with primary Sjogren's syndrome (SS) versus rheumatoid arthritis (RA) and healthy controls (HC). Increased IMT scores were detected in patients with primary SS or RA compared to HC (mean ± SD 1.0 ± 0.3 and 1.0 ± 0.4 versus 0.9 ± 0.2; *P* = 0.03 and *P* = 0.002, respectively).

**Figure 2:**
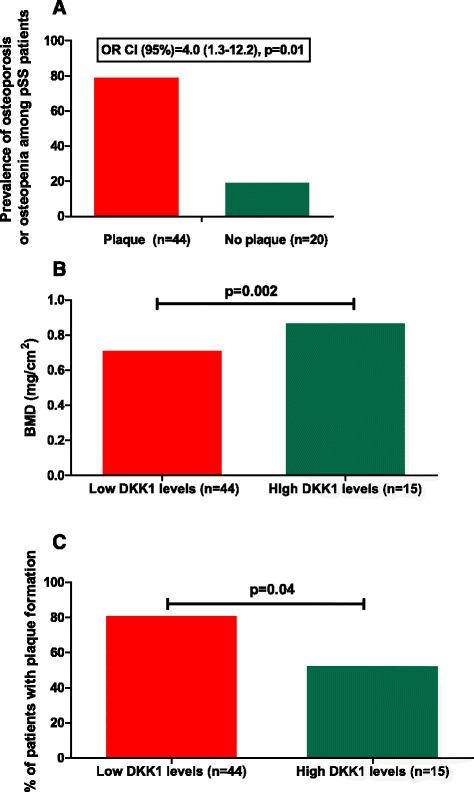
Independent association of osteopenia/osteoporosis (OP) with atherosclerotic plaque formation in patients with primary Sjogren's syndrome (SS). **(A)** Association of osteopenia/OP with plaque formation in SS (odds ratio (OR) 4.0, 95% CI 1.3, 12.2, *P* = 0.01). This association remained significant when potential confounders for both clinical entities, such as age, total steroid dose and smoking, were taken in consideration. **(B)** Patients with primary SS characterized by low Dickkopf-related protein 1 (DKK1) levels had reduced bone mass levels compared to those with high DKK1 levels (0.7 ± 0.1 versus 0.9 ± 0.1 mg/cm^2^, *P* = 0.002). **(C)** Similarly, patients with primary SS with low DKK1 levels had increased rates of plaque formation (81.8% versus 53.3%). The sum of the mean + 2 SD of healthy controls (HC) (2403.6 pg/ml) was considered as the cut off value for low and high DKK1 levels.

Sjogren’s syndrome (SS) or autoimmune epithelitis is a slowly progressing autoimmune disease characterized by salivary and lacrimal gland dysfunction, resulting in oral and ocular dryness. The syndrome shares many common clinical features and serologic markers with other immune-mediated autoimmune diseases, especially SLE [12,13]. In contrast to SLE, available data on comorbidities in SS are rather limited.

In the present study, we sought to determine the prevalence of subclinical atherosclerosis and impaired bone health (OP or osteopenia) in patients with primary SS and to explore whether they are associated with disease-related features and with traditional risk factors for cardiovascular disease (CVD) and OP. In addition, the Wingless-type (Wnt) signaling pathway, which has been implicated in the pathophysiology of both CVD and OP [14,15], was also explored.

## Methods

### Study subjects

In the present prospective cohort study, 64 consecutive patients with primary SS according to the American-European Classification Criteria [16], 77 patients with RA fulfilling the American College of Rheumatology classification criteria for RA and 60 consecutive healthy individuals (of similar age and sex distribution to the patients with primary SS) were studied as disease and healthy controls (HC), respectively. All patients were followed at the Department of Pathophysiology, School of Medicine, University of Athens and Department of Rheumatology, General Hospital of Athens G. Gennimatas. Exclusion criteria for all participants included pregnancy, age <18 years, renal dysfunction (serum creatinine levels >3 mg/dl, creatinine clearance <30 ml/minute). Serum was collected from patients and controls and stored at −70^°^C. The study has been approved by the Ethics Committee of Laikon General hospital and General Hospital of Athens G. Gennimatas. All patients provided written informed consent prior to their entry in the study.

### Clinical assessment

Demographic data, clinical features, therapeutic regimens and classical risk factors for atherosclerosis and OP were recorded in all participating patients and controls. Demographic data included age, sex, and BMI. Clinical manifestations included the presence of subjective and objective oral and ocular dryness (documented by unstimulated salivary flow rates and Schirmer’s test/Rose Bengal staining, respectively) [16]; dry cough; dyspareunia; fever; arthralgias; arthritis; carpal tunnel syndrome (documented by physical examination and nerve conduction studies); Raynaud's phenomenon; lymphadenopathy; splenomegaly; purpura; pulmonary involvement (small airway disease or interstitial lung disease documented by pulmonary function tests and high resolution computed tomography scans); pleuritis; pericarditis; renal involvement including interstitial nephritis (documented by urine-specific gravity <1.010 or pH >5.5 on at least two consecutive measurements after fluid restriction) and glomerulonephritis documented by renal biopsy; liver involvement (documented by liver biopsy showing changes compatible with primary biliary cirrhosis in the setting of increased liver enzymes or anti-mitochondrial antibodies); peri-epithelial disease (defined as peribronchial, interstitial nephritis, autoimmune cholangitis); myositis (documented by muscle biopsy in the setting of increased aldolase or creatinophosphokinase); peripheral neuropathy (documented by nerve conduction studies in patients with clinical symptoms or signs suggestive of neuropathy); central nervous involvement; lymphoma (documented by biopsy) and other neoplastic diseases. Sjogren’s syndrome disease activity index (ESSDAI) was also determined [17].

Classical risk factors for atherosclerosis and OP included the presence of family history of coronary disease (defined as a cardiovascular episode occurring below the age of 55 years in men and below 65 years in women in first-degree relatives); family history of low-energy fracture; past medical history (PMH) of coronary heart disease, stroke, DM, HT, low-energy fractures, malabsorption, or thyroid function abnormalities; smoking history; alcohol consumption (units/weeks); age of menarche and menopause (for women) and total/current steroid use.

### Hematological, biochemical and immunological parameters

Full blood count results, Westergren erythrocyte sedimentation rate (ESR), C-reactive protein (CRP) levels (cut off value 5 mg/l) and kidney, liver and thyroid function tests were recorded. Additionally, plasma levels of total cholesterol and high-density lipoprotein (HDL) cholesterol, low-density lipoprotein (LDL) cholesterol, triglyceride, homocysteine and fibrinogen levels were determined for all patients and controls, after overnight fasting. Serum and 24-hour urine calcium and phosphorus levels, intact parathyroid hormone (PTH) (pg/ml) and 25 (hydroxy) vitamin D (25(OH) D_3_) (ng/ml) levels were also measured.

Immunological profile testing included antinuclear antibodies (ANA), antibodies to Ro (SSA) and La (SSB) antigens, complement levels, rheumatoid factor (RF) and second-generation anti-cyclic citrullinated peptide antibody (only in RA patients); hypergammaglobulinemia (total gamma globulins >2 g/l) and cryoglobulins.

### Wnt signaling pathway

To determine whether the Wnt signaling pathway is implicated in the concomitant presence of CVD and impaired bone health in patients with primary SS, serum levels of Dickkopf-related protein 1 (DKK1) and sclerostin were also determined in available sera from 59 patients with primary SS and 17 HC by commercial ELISA, as per manufacturer instructions (R&D Systems, Minneapolis, MN, USA). According to these results, SS patients were further subdivided in those characterized by low and high DKK1 and sclerostin levels. As a cut off value was considered the sum of the mean + 2 SD of HC (for DKK1 2403.6 pg/ml and sclerostin levels 212.2 pg/ml).

### Assessment of subclinical atherosclerosis

The presence of subclinical atherosclerosis was defined by the presence of plaque and/or arterial wall thickening (defined as intima media thickness (IMT) score >0.90 mm in carotid and femoral arteries as determined by ultrasound (iU22, Philips, Royal Philips Electronics of the Netherlands)) [18]. Both the carotid (common carotid, bifurcation, and internal carotid) and the femoral (common femoral and superficial femoral) arteries were evaluated in each individual. The mean carotid artery IMT was defined as the average of 36 IMT readings (common, bifurcation, and internal carotid arteries, right and left side, far and near wall, with three sampling points per segment) and the mean femoral artery IMT was the average of twenty-four IMT readings (common and superficial femoral arteries, right and left side, far and near wall, with three sampling points per segment), as previously described [19]. Plaque formation was defined as a focal protrusion of more than 50% of the surrounding wall [20,21]. The radiologist (EA) in charge of the ultrasound scanning was unaware of the clinical diagnosis of the subjects under evaluation.

### Bone mineral density (BMD) measurements

BMD measurements were performed in all patients and controls by dual-energy x-ray absorptiometry using the QDR4500 densitometer (Hologic, Bedford, MA, USA), and were cross calibrated. On the basis of the individual’s age, BMD determination was performed either on the hip (total hip region) or the spine (anteroposterior lumbar spine, lumbar vertebrae L1 to L4), for ages above or below 65 years, respectively, according to previously published guidelines [22]. The presence of OP or osteopenia was defined according to the World Health Organization (WHO) classification system, as a T-score < −2.5 SD and −1 SD, respectively, at either the lumbar spine or the hip [23].

### Statistical analysis

Two-sided Fisher’s exact/chi-square and Mann–Whitney tests were implemented to compare qualitative and quantitative characteristics, respectively, between patient groups and HC (Graph Pad Prism 5.00, GraphPad Software, San Diego, CA, USA). In order to explore the independent contribution of SS itself to the increased IMT levels, a multivariate model was constructed including age, sex, HT, smoking (pack/years), LDL and HDL. Determinants of arterial wall thickening (defined as IMT >0.90 mm) and plaque, and the presence of OP or osteopenia in the setting of SS and RA were assessed by univariate and multivariate models. Multivariate models included the traditional risk factors for CVD or OP/osteopenia, and disease-related features that were found to be significant in univariate analysis. In order to test whether the presence of plaque formation was independently associated with the presence of OP/osteopenia in primary SS and RA, another model was constructed including well-established common risk factors for both plaque formation and impaired bone health such as age, total steroid dose and history of smoking. A *P*-value <0.05 for univariate analyses and <0.1 for multivariate analyses, respectively, were considered statistically significant. Data were stored in the SPSS statistical package.

## Results

### Demographics - traditional risk factors for CVD and OP

Table S1 summarizes the demographic characteristics and prevalence of traditional risk factors for CVD and OP in patients with primary SS or RA, and in age- and sex-matched HC (see Additional file 1). As shown, the age distribution and sex ratio did not differ significantly among the three groups. Patients with primary SS had a mean age of 57.2 ± 12.4 years, a female-to-male ratio of 15 to 1 and disease duration of 8.4 ± 7.0 (mean ± SD) years. Patients with RA had a mean age of 58.4 ± 11.3 years, a female-to-male ratio of 14.6 to 1.0 and disease duration of 14.8 ± 11.0 years, which was significantly higher compared to the primary SS group. The corresponding values for HC were 56.4 ± 7.8 years and the female-to-male ratio was 14 to 1.

In regard to traditional CV and OP risk factors, compared to the HC group, patients with primary SS had lower cholesterol levels and reduced rates of family history of CVD (mean ± SD 194.6 ± 34.2 mg/dl versus 211.8 ± 32.2 mg/dl; *P* = 0.01, 14.2% versus 28.8%; *P* = 0.04 respectively). Compared to the RA group, patients with primary SS had smaller disease duration (8.4 ± 7.03 versus 14.8 ± 11.0 years; *P* = 0.0004), reduced current daily steroid dose intake (1.3 ± 2.2 mg versus 4.8 ± 4.7 mg; *P* = 0.0001), lower serum triglyceride (103.7 ± 43.1 mg/dl versus 124.7 ± 53.8 mg/dl; *P* = 0.01) and CRP levels (4.6 ± 10.7 mg/l versus 14.8 ± 27.9 mg/l; *P* <0.0001) as well as higher 25(OH) vitamin D and phosphate levels (21.3 ± 11.4 ng/ml versus 17.5 ± 7.1 ng/ml; *P* = 0.04 and 3.3 ± 0.5 mg/dl versus 3.1 ± 0.5 mg/dl; *P* = 0.01, respectively). On the other hand, RA patients demonstrated increased rates of DM (14.1% versus 0%, *P* = 0.002), significantly elevated triglyceride levels (124.7 ± 53.8 mg/dl versus 104.8 ± 53.2 mg/dl; *P* = 0.001), lower serum phosphate and 25(OH)D3 levels (3.1 ± 0.5 mg/dl versus 3.4 ± 0.4 mg/dl; *P* = 0.0008, 17.5 ± 7.1 ng/ml versus 22.2 ± 7.1 ng/ml; *P* = 0.0009) and increased CRP levels compared to HC (14.8 ± 27.9 mg/l versus 2.7 ± 2.3 mg/l; *P* = 0.0004, respectively).

### Prevalence of comorbidities in patients with primary SS or RA and in age- and sex-matched healthy individuals

Increased IMT scores were detected in patients with primary SS and RA compared to HC (mean ± SD 1.0 ± 0.3 and 1.0 ± 0.4 versus 0.9 ± 0.2; *P* = 0.03 and *P* = 0.002, respectively) (Figure 1, Table 1). Additionally, the rates of arterial wall thickening (defined as IMT >0.90 mm), carotid and femoral plaque formation, OP/osteopenia and low-energy fractures are displayed in Table 1. The prevalence of arterial wall thickening was found to be increased in primary SS compared to the HC group (59.4% versus 40.4%, odds ratio (OR) 2.2, 95% CI 1.02, 4.55; *P* <0.05). Of interest, the presence of primary SS emerged as an independent risk factor for arterial wall thickening when traditional risk factors for CVD including age, sex, HT, smoking (pack/years), LDL and HDL levels were taken into account in a multivariate model (adjusted OR 2.8, 95% CI 1.04, 7.54). Similar results were obtained for RA patients compared to HC (data not shown).

Additionally patients with RA (but not with SS) differed from HC in regard to plaque formation, OP/osteopenia and fractures (84.4% versus 56.9, *P* <0.001, 72.2% versus 50.9, *P* <0.05 and 23.9% versus 8.5, *P* <0.05, respectively). Compared to patients with primary SS, those with RA exhibited significantly higher rates of plaque formation and OP (84.4 versus 68.8, *P* <0.05 and 18.1% versus 7.8%, *P* <0.05, respectively).

### Associations between IMT levels and traditional risk factors for CVD and disease-related features in patients with primary SS

We next wished to identify predictors of arterial wall thickening in the setting of primary SS. In the primary SS group, a statistically significant association was observed between IMT levels and traditional CVD risk factors including age, BMI, HT, LDL and uric acid levels. Among disease-related features, the presence of peri-epithelial disease, increased levels of C3 and decreased urine specific gravity were also found to be significantly associated with increased IMT scores (see Table 2). Multivariate analysis revealed only age as independent predictor of arterial wall thickening in the setting of primary SS (OR 1.1, 95% CI 1.1, 1.2; *P* = 0.002). No statistical significant associations were detected between total steroid dose and IMT score in primary SS (*r* = −0.07; *P* = 0.57, Spearman’s rank correlation).

### Predictors of plaque formation in patients with primary SS

The presence of carotid and/or femoral artery plaque in the univariate analysis was positively associated with age, the focus score on minor salivary gland biopsy and Raynaud’s phenomenon, and was negatively associated with whole salivary flow rates and lymphocytic numbers (Table 3). Multivariate analysis confirmed age and absolute number of lymphocytes as independent predictors of plaque formation in the context of primary SS (OR 1.1, 95% CI 1.0, 1.1 and OR 0.9, 95% CI 0.9, 1.0, respectively).

### Determinants of OP and/or osteopenia in patients with primary SS

In univariate analysis the classical risk factors of age and serum calcium levels were identified to be positively associated with the presence of OP and/or osteopenia in patients with primary SS. Additionally, patients with evidence of either osteopenia or OP exhibited lower rates of a positive family history of fracture, increased prevalence of arthralgia and higher mean values of urine pH. In multivariate analysis serum calcium and increased urine pH emerged as independent predictors of OP or osteopenia among patients with primary SS (OR 3.2, 95% CI 1.01, 10.4) and OR 4.5, 95% CI 1.004, 20.4, respectively) (Table 4).

Further analysis of the prevalence of OP and/or osteopenia was subsequently carried out by subdividing the patients with primary SS according to the urinary pH levels (cut off 5.5). According to this subgroup analysis, the patients with primary SS who had urinary pH >5.5 at the first visit exhibited significantly increased rates of OP or osteopenia compared to those with urinary pH <5.5 (94.3% versus 44.4%, *P* = 0.002) (Additional file 2: Figure S1).

In a subset of 57 patients with primary SS, urine levels of type 1 cross-linked c-telopeptide (CTX) were determined; no statistical significant associations were detected between CTX levels, BMD levels, total steroid dose and SS-activity index as analyzed by Spearman’s rank correlation (data not shown).

### Independent association of impaired bone health with atherosclerotic plaque formation in patients with primary SS

Given the previously reported associations between alterations in bone mass with atherosclerotic risk in healthy populations, we sought to explore similar associations in an autoimmune population, such as patients with primary SS [24,25]. In fact, the presence of either OP or osteopenia in our SS study group was found to be strongly associated with the presence of plaque, as detected by carotid and femoral ultrasound (OR 4.0, 95% CI 1.3, 12.2; *P* = 0.01) with 80% of patients with plaque having evidence of impaired bone health versus 20% of those with no plaque (Figure 2A). This association remained significant when classical predictors of both clinical entities, such as age, total steroid dose and smoking, were included in the multivariate model.

In order to investigate whether the Wnt signaling system was involved in the concomitant occurrence of both atherosclerotic and osteoporotic disease in our SS patients, DKK1 and sclerostin levels were also determined by commercial ELISA. A positive correlation between sclerostin and DKK1 levels with neck BMD levels was detected (*r* = 0.42; *P* = 0.02 and *r* = 0.41; *P* = 0.01, respectively). However, no association was found between sclerostin levels and the presence of atherosclerotic plaque formation (data not shown). On the other hand, patients with primary SS characterized by low DKK1 levels compared to those with high DKK1 levels had reduced bone mass levels (0.7 ± 0.1 versus 0.9 ± 0.1 mg/cm^2^) and increased rates of plaque formation (81.8% versus 53.3%), implying Wnt signaling as a potential contributor for the concomitant presence of both comorbidities in a subgroup of patients with primary SS (Figure 2B and C).

### Associations between IMT levels and plaque formation with traditional risk factors for CVD and disease-related features in RA patients

In univariate analysis, arterial wall thickening among RA patients was identified to be positively associated with age, BMI, triglyceride levels, number of tender joints and blood urea nitrogen (BUN) levels, and negatively associated with cyclosporine treatment, CRP levels and number of platelets/mm^3^. Age, BMI, the number of tender joints and cyclosporine use were identified as independent predictors of arterial wall thickening in the setting of RA in multivariate analysis (see Additional file 1: Table S2). No statistically significant associations were detected between total steroid dose and IMT score in the RA population (*r* = 0.20; *P* = 0.89, Spearman’s rank correlation).

RA patients with carotid and/or femoral artery plaque were older, with higher BMI, higher cumulative steroid doses and BUN levels and increased rates of HT compared to those without plaque formation. Paradoxically, plaque formation was negatively associated with history of smoking among RA patients in univariate analysis (see Additional file 1: Table S3). Multivariate analysis confirmed age and total steroid dose as independent predictors of plaque formation in the context of RA.

### Determinants of OP and/or osteopenia in RA patients

In both univariate and multivariate analysis, increased age, PMH of fracture and increased PTH were identified to be positively associated with the presence of OP and/or osteopenia in RA patients (see Additional file 1: Table S4).

## Discussion

In the present study we wished first to determine the prevalence of comorbidities such as subclinical atherosclerosis and impaired bone health in a cohort of patients with primary SS compared to healthy and RA individuals with a similar age/sex distribution, and second, to explore potential associations with disease-related features and traditional risk factors for CVD and OP. We have shown that the presence of primary SS was as an independent risk factor for arterial wall thickening, when traditional risk factors for CVD including age, sex, HT, smoking (pack/years), LDL and HDL levels were taken into account in a multivariate model. While the carotid/femoral ΙΜΤ levels were found to be significantly higher in primary SS and RA patients versus HC, only RA patients demonstrated increased rates of plaque formation compared to both SS patients and HC. The higher proportion of DM and the increased triglyceride and CRP levels, together with the lower vitamin D levels observed in the RA cohort could account for the pronounced atherosclerotic risk in these patients, as previously reported [26-28].

We next aimed to explore whether disease-related and/or traditional risk factors could account for the increased rates of comorbidities among patients with primary SS. Advanced age, higher hypertension rates and BMI, increased LDL and uric acid levels and the presence of peri-epithelial disease were found to be associated with arterial wall thickening, while advanced age, higher focus score on minor salivary gland biopsy and reduced salivary flow rates, the presence of Raynaud’s syndrome and reduced lymphocytic numbers were predictors of plaque formation. In multivariate models, age was an independent predictor of both arterial wall thickening and plaque formation, and lymphopenia was an independent risk factor for plaque formation. Although increased prevalence of DM [29], hypertriglyceridemia [29,30], HT [30] and dyslipidemia [31,32] were previously reported to be higher among SS populations, we have not observed significant differences between patients with primary SS and HC, except for lower rates of family history of CVD and cholesterol levels in the primary SS group. The latter observation was also supported by Lodde *et al*. [33].

Although the increased atherosclerotic risk is well-established in patients with SLE [34], RA [35] and systemic sclerosis [36], a limited body of evidence is available on primary SS syndrome. Vaudo *et al*., observed increased carotid and femoral IMT scores in white women with primary SS compared to age- and sex-matched HC in association with leucopenia and the presence of anti-SSA antibodies [18,19]. Although no association with anti-Ro/SSA antibodies and IMT values was detected in our cohort, we also revealed lymphopenia as a significant predictor of plaque formation in our patient group. Additionally, endothelial dysfunction identified by abnormal ankle brachial index (ABI), lower endothelium-dependent flow-mediated vasodilatation (FMV), increased asymmetric dimethylarginine (ADMA) and higher pulse wave velocity (PWV) values were recently demonstrated [37-39]. At the time of preparation of the current manuscript, data from a large multicenter Italian study demonstrated an increased risk of cerebrovascular events and myocardial infarction among primary SS populations [32]. On the other hand, impaired smooth-muscle relaxation was present in patients with primary SS independent of endothelial contribution, and particularly in those characterized by leucopenia, RF, anti-SSB antibodies and articular involvement [38].

The mechanisms for the observed atherosclerotic risk in SS have not yet been elucidated. Data derived mainly from lupus studies suggest that T and B cell dysregulation heightens production of cytokines such as inteleukin-17 (IL-17), interferon γ (ΙFNγ), and more recently interferon α (ΙFNα), a central cytokine in both lupus and SS pathogenesis, which have been all considered to contribute to the increased cardiovascular risk of these patients. Particularly, impairment of endothelial repair through accelerated apoptosis of endothelial progenitor cells, together with enhancement of foam cell formation in the atherosclerotic plaque, have been proposed to account for the atherogenic potential of IFNα [40].

Age, BMI and clinical variables of disease activity as evidenced by increased number of tender joints were independent predictors of increased IMT scores in the RA group, whereas cyclosporine use had a protective role against arterial wall thickening among these patients, in line with previous observations in SLE patients, in which use of cyclosporine A protected against increased carotid IMT, leading to a decreased risk of arteriosclerosis [41]. Plaque formation was independently associated with age and total steroid dose, in line with previous observations [42].

Although an increased risk of OP and fracture rates in autoimmune diseases such as SLE [9], RA [43] and systemic sclerosis [11] is well-documented, no information is available on the occurrence of OP/osteopenia or fracture rates in patients with primary SS. Patients with primary SS share a number of clinical and serological features which theoretically predispose to low bone mineralization; these include low vitamin D levels, hypercalciuria related to underlying interstitial nephritis, steroid use for systemic involvement, and coexistence with other organ-specific autoimmune disorders known to increase OP risk, such as primary biliary cirrhosis and celiac disease [44-46]. In our hands, the rate of positive antimitochondrial as well as antigliandin and antitransglutaminase antibodies, respectively, was low, and therefore firm conclusions cannot be drawn (data not shown). In regard to OP/osteopenia and fracture rates and in contrast with RA patients, no statistically significant differences were observed between primary SS group as a whole and the HC group. It was surprising that patients with primary SS experienced paradoxically higher rates of fractures compared to HC, despite the lower rates of osteopenia. Given that the differences noted were not statistically significant, we presume that this was a chance finding.

When patients with primary SS were stratified according to urine pH, which was an independent predictor of OP and or/osteopenia in the multivariate analysis, significantly increased prevalence of OP/osteopenia was observed compared to the controls. Although vitamin D and serum phosphate levels were decreased in RA patients compared to HC, which might contribute to the increased rates of OP and fracture risk in this population, they were within normal limits within our SS cohort. Additionally, mean current steroid dose and CRP levels as a marker of systemic inflammation, which was previously implicated in osteoporotic risk among RA patients [47], were increased in our RA cohort, in which independent predictors of OP/osteopenia included PMH of fracture, increased serum levels of PTH and advanced age, as previously reported [48].

A growing body of evidence over the last decade supports the association between bone mass reduction and atherosclerotic risk in healthy populations [49,50]. In the present study, we also observed the concomitant occurrence of decreased bone mass and plaque formation in our autoimmune SS group, when potential confounders for both clinical entities, such as age, total steroid dose and smoking were considered. Such an association was not detected in our RA cohort, possibly related to the confounding effects of concomitant medications.

To further explore the underlying linking mechanisms between impaired bone health and atherosclerotic risk in primary SS, inhibitors of the Wnt signaling pathway were measured, including sclerostin and DKK1, which were previously potentially implicated in the pathogenesis of arterial calcification and/or inhibition of bone formation [51-53]. In accordance with previously reported findings in diabetic [52] and in predialysis patients with chonic kidney disease [54], an inverse relationship was identified between the levels of DKK1 and presence of plaque formation in patients with primary SS. On the other hand, BMD was positively associated with serum sclerostin, as already suggested, as well as with DKK1 levels, an observation not previously reported [14]. A plausible scenario for the current observations could be that osteoporotic/osteopenic SS patients, characterized by suppressed Wnt signaling, display paradoxically reduced DKK1 levels possibly due to the absence of a negative feedback provided by Wnt inhibitors as previously suggested [14]. As a result, a lack of DKK1-mediated protection against vascular wall calcification could lead to plaque formation and increased atherosclerotic risk.

## Conclusion

In conclusion, in the present study we demonstrated that almost two thirds of patients with primary SS have evidence of subclinical atherosclerosis and impaired bone health, which may be attributed in part to the presence of traditional risk factors as well as disease-related features, with Wnt pathway mediators possibly accounting for this observation. Further larger studies are warranted to better explore the contribution of the Wnt signaling pathway in the pathogenesis of these comorbidities in both healthy and autoimmune populations.

## Additional files

Additional file 1:
**Table S1.** Characteristics of primary SS and rheumatoid arthritis (RA) patients and age-sex matched healthy individuals. **Table S2.** Traditional and disease-related predictors of arterial wall thickening in patients with RA. **Table S3.** Traditional and disease-related predictors of plaque formation in patients with RA. **Table S4.** Determinants of osteoporosis or osteopenia in RA patients. Additional file 2: Figure S1. Increased rates of osteoporosis/osteopenia in patients with primary Sjogren’s syndrome (SS) with urine pH >5.5.

## Abbreviations

ANA: antinuclear antibodies
BMD: bone mineral density
BMI: body mass index
CRP: C-reactive protein
CTX: type 1 cross-linked c-telopeptide
CVD: cardiovascular disease
DKK1: Dickkopf-related protein 1
DM: ciabetes mellitus
ELISA: enzyme-linked immunosorbent assay
ESR: erythrocyte sedimentation rate
ESSDAI: Sjogren’s syndrome disease activity index
HC: healthy controls
HDL: high-density lipoprotein
HT: hypertension
IFN: Interferon
IL-17: Interleukin-17
IMT: intima media thickness
LDL: low-density lipoprotein
OH: hydroxy
OP: osteoporosis
OR: odds ratio
PMH: past medical history
PTH: parathyroid hormone
RA: rheumatoid arthritis
RF: rheumatoid factor
SLE: systemic lupus erythematosous
SS: Sjogren’s syndrome
SSA: antibodies to Ro
SSB: antibodies to La
WHO: World Health Organization
Wnt: Wingless-type
