# Supplementary material for: Subclinical atherosclerosis and impaired bone health in patients with primary Sjogren’s syndrome: prevalence, clinical and laboratory associations
Source: Arthritis Res Ther. 2015 Apr 11;17(1):99. doi: 10.1186/s13075-015-0613-6 (PMC4416325; doi:10.1186/s13075-015-0613-6)
Supplement: Additional file 1: — Table S1. Characteristics of primary SS and rheumatoid arthritis (RA) patients and age-sex matched healthy individuals. Table S2. Traditional and disease-related predictors of arterial wall thickening in patients with RA. Table S3. Traditional and disease-related predictors of plaque formation in patients with RA. Table S4. Determinants of osteoporosis or osteopenia in RA patients. [file 13075_2015_613_MOESM1_ESM.doc]

**Supplementary Table 1:** Characteristics of primary SS and RA patients and age-sex matched healthy individuals.

|  | **SS** | **RA** | **HC** | **p-value*** | **p-value**** | **p-value***** |
| --- | --- | --- | --- | --- | --- | --- |
| **Age(yrs)** | 57.2±12.4 | 58.4±11.3 | 56.4±7.8 | ns | ns | ns |
| **Sex (F:M ratio)** | 15/1 | 14.6/1 | 14/1 | ns | ns | ns |
| **Disease duration** | 8.4±7.0 | 14.8±11.0 | Na | 0.0004 | na | na |
| **PMH of CVD (%)** | 6.3 | 5.12 | 0 | ns | ns | ns |
| **FH of CVD (%)** | 14.2 | 25.6 | 28.8 | ns | 0.04 | ns |
| **Smoking(packs/year)** | 7.7±16.9 | 14.3±46.3 | 4.7±10.0 | ns | ns | ns |
| **% of females in menopause** | 76.6 | 74 | 90.5 | ns | ns | ns |
| **BMI** | 27.4±5.5 | 28.3±5.8 | 26.5±3.7 | ns | ns | ns |
| **Diabetes (%)** | 6.3 | 14.1 | 0 | ns | ns | 0.002 |
| **Hypertension ( %)** | 36.5 | 33.3 | 25.8 | ns | ns | ns |
| **Cholesterol levels (mg/dl)** | 194.6±34.2 | 201.8±37.8 | 211.8±32.2 | ns | 0.01 | ns |
| **HDL(mg/dl)** | 54.9±14.5 | 56.4±16.2 | 58.1±14.6 | ns | ns | ns |
| **LDL(mg/dl)** | 120.4±30.2 | 121±28.5 | 132.8±29.7 | ns | 0.04 | 0.02 |
| **Triglycerides (mg/dl)** | 103.7±43.1 | 124.7±53.8 | 104.8±53.2 | 0.01 | ns | 0.001 |
| **Homocysteine levels (μmol/L)** | 14.4±5.2 | 13.7±5.7 | 12.9±3.2 | ns | ns | ns |
| **Uric acid(mg/dl)** | 4.1±1.2 | 4.1±1.6 | 3.7±1.1 | ns | ns | ns |
| **Ca (mg/dl)** | 9.5±0.5 | 9.6±0.5 | 9.6±0.4 | ns | ns | ns |
| **PO4(mg/dl)** | 3.3±0.5 | 3.1±0.5 | 3.4±0.4 | 0.01 | ns | 0.0008 |
| **Ca urine 24 hrs (gr)** | 0.1±0.1 | 0.1±0.1 | 0.1±0.1 | ns | ns | ns |
| **PTH(pg)/ml** | 52.9±18.4 | 51.3±19.7 | 52.2±17.8 | ns | ns | ns |
| **25-hydroxy vitamin D3(ng/ml)** | 21.3±11.4 | 17.5±7.1 | 22.2±7.1 | 0.04 | ns | 0.0009 |
| **Total steroid dose (g)** | 7.38±14.9 | 0.94±1.4 | na | ns | na | na |
| **Current steroid dose mg** | 1.3±2.2 | 4.8±4.7 | na | 0.0001 | na | na |
| **Current TSH levels (μ ΙU /dl)** | 1.6±1.4 | 1.8±1.2 | 1.9±1.4 | ns | ns | ns |
| **CRP (mg/l)** | 4.6±10.7 | 14.8±27.9 | 2.7±2.3 | <0.0001 | ns | 0.0004 |
| **Fibrinogen (mg/dl )** | 407.9±116.1 | 440.9±137.4 | 360.2±66.4 | ns | ns | 0.002 |

*p-value: pSS vs RA,**p-value: pSS vs HC ,***p-value: RA vs HC

| **TRADITIONAL RISK FACTORS** | **IMT>0.90 mm**  **(n=46)** | **IMT≤0.90 mm (n=27)** | **p-value** | **OR 95%(CI)** | **p-value** |
| --- | --- | --- | --- | --- | --- |
| Age(yrs) | 62.0±9.2 | 53.9±11.8 | 0.004 | 1.1 (1.0-1.2) | 0.04 |
| % of females | 95.7 | 85.2 | ns |  |  |
| PMH of CVD (%) | 8.7 | 0.0 | ns |  |  |
| FH of CVD (%) | 30.4 | 18.5 | ns |  |  |
| Smoking (%) | 23.9 | 22.2 | ns |  |  |
| BMI | 29.6±6.0 | 26.3±5.3 | 0.02 | 1.1 (1.0-1.3) | 0.05 |
| Diabetes (%) | 19.6 | 7.4 | ns |  |  |
| Hypertension ( %) | 41.3 | 22.2 | ns |  |  |
| Cholesterol levels (mg/dl) | 205.2±36.4 | 195.5±40.2 | ns |  |  |
| HDL(mg/dl) | 54.7±16.3 | 57±15.3 | ns |  |  |
| LDL(mg/dl) | 124.0±28.9 | 116.6±27.7 | ns |  |  |
| Triglycerides (mg/dl) | 136.9±52.1 | 110.2±55.2 | 0.02 |  |  |
| Homocysteine levels (μmol/L) | 14.3±5.9 | 13.3±5.9 | ns |  |  |
| Uric acid(mg/dl) | 4.3±1.8 | 4.0±1.3 | ns |  |  |
| Current TSH levels (μ ΙU /dl) | 1.7±1.3 | 1.9±1.1 | ns |  |  |
| 25-hydroxy vitamin D3(ng/ml) | 17.7±6.7 | 17.6±8.1 | ns |  |  |
| **DISEASE RELATED FEATURES** |  |  |  |  |  |
| Disease duration (years) | 15.5±10.9 | 13.6±10.6 | ns |  |  |
| DAS 28 | 4.6±1.4 | 4.0±1.4 | ns |  |  |
| Swollen joints | 3.7±4.3 | 3.2±3.3 | ns |  |  |
| Tender joints | 6.0±5.9 | 3.4±4.0 | 0.02 | 1.2 (1.0-1.4) | 0.05 |
| Rheumatoid factor titers (IU/ml) | 143.1±335.5 | 188.6±462.6 | ns |  |  |
| Anti-citrullinated antibodies (IU/ml) | 161.3±171.1 | 143.4±137.9 | ns |  |  |
| Hemoglobin levels (gr/dl) | 13.7±4.8 | 12.7±1.5 | ns |  |  |
| Platelets (/μL) | 279,304±77,780 | 329,518±123,222 | 0.04 |  |  |
| BUN (mg/dl ) | 41.8±13.9 | 34.7±9.4 | 0.02 |  |  |
| ESR (mm/hr) | 32.9±24.4 | 35.3±25.7 | ns |  |  |
| CRP (mg/l) | 10.0±16.8 | 24.0±41.6 | 0.04 |  |  |
| Fibrinogen (mg/dl) | 413.9±105.4 | 478.7±177.0 | ns |  |  |
| **MEDICATIONS** |  |  |  |  |  |
| Current steroid dose (mg/d) | 4.0±3.05 | 5.9±6.2 | ns |  |  |
| Total steroid dose (mg) | 1,003 ±1,442 | 955±1,402 | ns |  |  |
| Methotrexate (%) | 74.4 | 74.1 | ns |  |  |
| Leflunomide (%) | 37.0 | 30.8 | ns |  |  |
| Cyclosporine (%) | 11.4 | 37.0 | 0.02 | 0.2 (0.1-1.0) | 0.05 |
| Infliximab (%) | 32.6 | 37.0 | ns |  |  |
| Etanercept (%) | 45.7 | 33.3 | ns |  |  |
| Anakinra (%) | 13.0 | 19.0 | ns |  |  |
| Tocilizumab (%) | 10.9 | 0.0 | ns |  |  |
| Abatacept (%) | 10.9 | 0.0 | ns |  |  |

**Supplementary table 2:** Traditional and disease related predictors of arterial wall thickening in patients with RA.

Multivariate: all significant in u

RA: Rheumatoid arthritis, IMT: Intima media thickness, OR 95% (CI): Odds Ratio 95% (Confidence Intervals), PMH: Past Medical History, CVD: Cardiovascular Disease, FH: Family History, BMI: Body Mass Index, HDL: High Density Lipoprotein, LDL: Low density Lipoprotein, , BUN: blood urea nitrogen, TSH: Thyroid stimulating hormone, CRP: C-Reactive protein, DAS: disease activity index, ESR: Erythrocyte sedimentation rate.

**Supplementary table 3:** Traditional and disease related predictors of plaque formation in patients with RA.

RA: Rheumatoid arthritis, OR 95%(CI): Odds Ratio 95% (Confidence Intervals), PMH: Past Medical History, CVD: Cardiovascular Disease, FH: Family History, BMI:Body Mass Index, HDL: High Density Lipoprotein, LDL: Low density Lipoprotein, TSH: Thyroid stimulating hormone, CRP: C-Reactive protein, DAS: disease activity index, BUN: blood urea nitrogen, ESR: Erythrocyte sedimentation rate.

| **TRADITIONAL RISK FACTORS** | **Plaque**  **(n=65)** | **Absence of Plaque**  **(n=12)** | **p-value** | **OR 95%(CI)** | **p-value** |
| --- | --- | --- | --- | --- | --- |
| Age(yrs) | 61.0±9.5 | 44.7±10.9 | <0.001 | 1.2 (1.1-1.4) | 0.003 |
| % of females | 93.8 | 83.3 | ns |  |  |
| PMH of CVD (%) | 6.2 | 0.0 | ns |  |  |
| FH of CVD (%) | 26.2 | 16.7 | ns |  |  |
| Smoking (%) | 18.5 | 66.7 | 0.001 |  |  |
| BMI | 29.0±5.8 | 24.5±4.3 | 0.009 |  |  |
| Diabetes (%) | 16.9 | 0.0 | ns |  |  |
| Hypertension ( %) | 38.5 | 8.3 | 0.05 |  |  |
| Cholesterol levels (mg/dl) | 202.6±38.9 | 194.9±31.5 | ns |  |  |
| HDL(mg/dl) | 55.8±16.5 | 56.4±10.1 | ns |  |  |
| LDL(mg/dl) | 122.3±29.2 | 114.6±26.1 | ns |  |  |
| Triglycerides (mg/dl) | 125.9±55.3 | 119.8±49.2 | ns |  |  |
| Homocysteine levels (μmol/L) | 14.2±6.1 | 11.6±2.4 | ns |  |  |
| Uric acid(mg/dl) | 4.2±1.7 | 3.7±0.9 | ns |  |  |
| Current TSH levels (μ ΙU /dl) | 1.8±1.2 | 1.9±1.1 | ns |  |  |
| 25-hydroxy vitamin D3(ng/ml) | 18.1±7.2 | 14.3±5.1 | ns |  |  |
| Osteoporosis or osteopenia (%) | 69.2 | 75.0 | ns |  |  |
| **DISEASE RELATED FEATURES** |  |  |  |  |  |
| Disease duration (years) | 15.4±11.2 | 12.0±10.6 | ns |  |  |
| DAS 28 | 4.4±1.5 | 4.2±0.6 | ns |  |  |
| Swollen joints | 3.8±4.2 | 2.1±0.9 | ns |  |  |
| Tender joints | 5.4±5.8 | 4.0±2.5 | ns |  |  |
| Rheumatoid factor (%) | 63.1 | 75 | ns |  |  |
| Anti-citrullinated antibodies (%) | 61.5 | 83.3 | ns |  |  |
| Hemoglobin levels | 13.3±4.1 | 12.7±1.8 | ns |  |  |
| Platelets (/mm^3^) | 298,892±102,493 | 308,416±67,660 | ns |  |  |
| BUN (mg/dl ) | 39.9±13.2 | 31.7±6.8 | 0.03 |  |  |
| ESR (mm/hr) | 35±25.6 | 28.1±15.9 | ns |  |  |
| CRP (mg/l) | 16.3±30.0 | 7.9±10.9 | ns |  |  |
| Fibrinogen (mg/dl) | 450±140 | 408±123 | ns |  |  |
| **MEDICATIONS** |  |  |  |  |  |
| Current steroid dose (mg) | 4.9±4.7 | 4.9±4.9 | ns |  |  |
| Total steroid dose mg | 1060±1481 | 359±383 | 0.002 | 1.003 (1-1.005) | 0.03 |
| Methotrexate (%) | 78.5 | 83.3 | ns |  |  |
| Leflunomide (%) | 35.9 | 33.3 | ns |  |  |
| Cyclosporine (%) | 22.2 | 8.3 | ns |  |  |
| Infliximab (%) | 35.4 | 25.0 | ns |  |  |
| Etanercept (%) | 40 | 50 | ns |  |  |
| Anakinra (%) | 17.2 | 0.0 | ns |  |  |
| Tocilizumab (%) | 7.7 | 0.0 | ns |  |  |
| Abatacept (%) | 7.7 | 0.0 | ns |  |  |
| Rituximab (%) | 23.1 | 25.0 | ns |  |  |

**Supplementary Table 4:** Determinants of osteoporosis or osteopenia in RA patients.

RA: Rheumatoid arthritis, OR 95%(CI): Odds Ratio 95% (Confidence Intervals), PMH: Past Medical History, FH: Family History, BMI:Body Mass Index, PTH: Parathyroid hormone levels, TSH: Thyroid stimulating hormone, DAS: disease activity index, ESR: Erythrocyte sedimentation rate, CRP: C-Reactive protein.

|  | **UNIVARIATE**  **ANALYSIS** | | | | **MULTIVARIATE**  **ANALYSIS*** | |
| --- | --- | --- | --- | --- | --- | --- |
|  | **Presence of**  **osteoporosis or osteopenia** | | **Absence of osteoporosis or osteopenia** | **p-value** | **OR 95% (CI)** | **p-value** |
| **CLASSICAL RISK FACTORS**  **FOR OSTEOPOROSIS** |  | | | | | |
| Age(yrs) | 60.2±10.5 | | 53.9±11.8 | <0.05 | 1.06(1.0-1.1) | 0.05 |
| % of females | 92.3 | | 95 | ns |  |  |
| PMH of fracture(%) | 31.4 | | 5.0 | 0.03 | 10.7(1.05-1.1) | 0.05 |
| FH of fracture (%) | 19.6 | | 21.1 | ns |  |  |
| BMI | 28.3±5.9 | | 28.4±6.0 | ns |  |  |
| Smoking (%) | 21.2 | | 35 | ns |  |  |
| Age of menarche | 13.2±1.7 | | 12.5±1.7 | ns |  |  |
| Age of menopause | 47.1±6.9 | | 47.5±5.1 | ns |  |  |
| Ca (mg/dl) | 9.5±0.5 | | 9.7±0.5 | 0.05 |  |  |
| P (mg/dl) | 3.2±0.5 | | 3.1±0.6 | ns |  |  |
| Ca urine/24hs (gr) | 0.2±0.2 | | 0.1±0.1 | ns |  |  |
| 25-hydroxy vitamin D3(ng/ml) | 17.7±7.6 | | 17.8±5.8 | ns |  |  |
| PTH(pg)/ml | 54.5±20.1 | | 42.3±16.3 | 0.01 | 1.04 (1.0-1.1) | 0.02 |
| Current TSH levels(μ IU/dl) | 1.8±1.3 | | 1.6±1.2 | ns |  |  |
| Total steroid dose (mg) | 1058.9±1568.4 | | 781.7±989.5 | ns |  |  |
| Current steroid dose (mg/d) | 4.7±4.7 | | 5.8±4.9 | ns |  |  |
| **DISEASE RELATED FEATURES** |  | | | | | |
| Disease duration (years) | 16.3±11.5 | 10.3±8.0 | | 0.05 |  |  |
| DAS 28 | 4.4±1.3 | 4.7±1.3 | | ns |  |  |
| Swollen joints | 3.7±3.8 | 3.3±4.2 | | ns |  |  |
| Tender joints | 5.2±5.1 | 6.0±6.4 | | ns |  |  |
| Rheumatoid factor (%) | 61.5 | 75 | | ns |  |  |
| Anti-citrullinated antibodies (%) | 63.5 | 70 | | ns |  |  |
| ESR (mm/hr) | 33.8±25.0 | 38.4±23.7 | | ns |  |  |
| CRP (mg/l) | 33.5±4.8 | 9.8±9.9 | | ns |  |  |
| Fibrinogen ( mg/dl ) | 443±143 | 469±122 | | ns |  |  |
