# Supplementary material for: Subclinical atherosclerosis and impaired bone health in patients with primary Sjogren’s syndrome: prevalence, clinical and laboratory associations
Source: Arthritis Res Ther. 2015 Apr 11;17(1):99. doi: 10.1186/s13075-015-0613-6 (PMC4416325; doi:10.1186/s13075-015-0613-6)
Supplement: Additional file 2: Figure S1. — Increased rates of osteoporosis/osteopenia in patients with primary Sjogren’s syndrome (SS) with urine pH >5.5. [file 13075_2015_613_MOESM2_ESM.docx]

**Supplementary figure 1: Increased rates of osteoporosis/osteopenia in primary SS patients with urine pH>5.5.**
